# Cilazapril-induced pleural effusion: A case report and review of the literature

**DOI:** 10.4103/1817-1737.65043

**Published:** 2010

**Authors:** Elif Kupeli, Gaye Ulubay, Sevinc Sarinc Ulasli, Dalokay Kilic

**Affiliations:** *Department of Chest Diseases, Baskent University School of Medicine, Ankara, Turkey*; 1 *Department of Thoracic Surgery, Baskent University School of Medicine, Ankara, Turkey*

**Keywords:** Angiotensin-converting-enzyme inhibitor, cilazapril, cilazapril-induced pleural effusion, lymphocytic pleural effusion

## Abstract

We describe an unusual case of lymphocytic pleural effusion associated with the use of cilazapril, a novel angiotensin-converting-enzyme inhibitor (ACEI). An 80-year-old male was prescribed cilazapril for hypertension. He subsequently presented with right chest pain and dry cough. He was found to have a lymphocytic pleural effusion on thoracentesis. Extensive workup, including open pleural biopsy, failed to reveal the etiology of the effusion. However, soon after the withdrawal of cilazapril, his clinical symptoms improved and the effusion disappeared. ACEI-induced pleural effusion has only been rarely reported. Drug-induced pleural effusion should be considered when formulating the differential diagnosis in a patient receiving ACEI.

Drug-induced pleural effusion (PE) is uncommon. The presumed mechanisms for the effusion include hypersensitivity or allergic reaction, direct toxic effect, oxygen free radical production, suppression of antioxidant defense, chemical pleuritis, and immune modulation.[1,2] Drugs are also known to cause a lupus-like syndrome that can manifest with PE.[3]

Angiotensin-converting-enzyme inhibitor (ACEI) causing PE is rare, only two cases having been reported.[2,4] We describe, what we believe is the first case of cilazapril (a novel ACEI)–induced lymphocytic PE.

## Case Report

An 80-year-old male, an ex-smoker, was admitted with right sided chest pain and dry cough of 6 weeks duration. He admitted to a short period of exposure to asbestos. Five years earlier he had received cilazapril 2.5 mg/day for treatment of hypertension but had had discontinued treatment for unknown reasons. The same drug was re-advised 6 months ago at the same dose and has been taking it since then. The patient had received no other medications.

On examination, there was dullness over the right hemi-thorax extending up to the angle of the scapula, along with corresponding reduction in the breath sounds.

**Figure 1:**
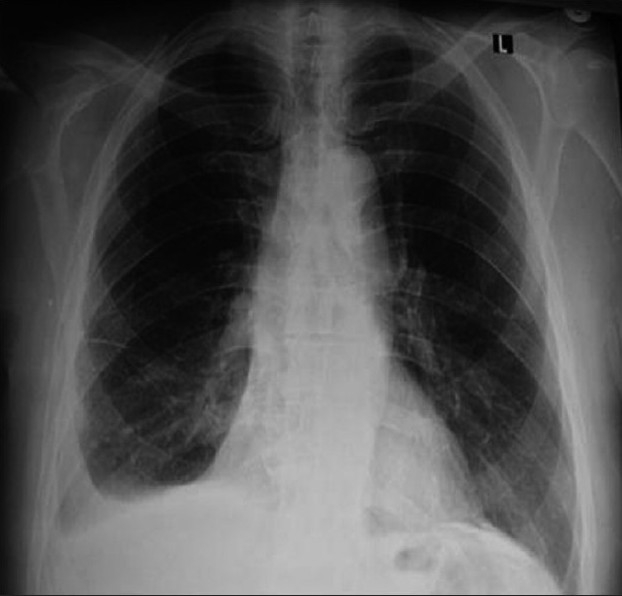
Posteroanterior chest X-ray shows minimal right-sided pleural effusion; there is left-sided pleural calcification

His white blood cell count with differential; hemoglobin; liver, renal and thyroid function tests; anti-nuclear anticore titers; rheumatoid factor; C-reactive proteins; erythrocyte sedimentation rate; d-dimer levels; and electrocardiogram were normal. Chest X-ray (CXR) revealed right effusion and left pleural calcification [Figure 1]. Computed tomography confirmed the effusion. There was no reason to suspect pulmonary embolism. Echocardiogram revealed a normal ejection fraction (54%) and ventricular function. Positron emission tomography (PET) revealed no hypermetabolic activity.

At thoracentesis, 20 ml of serosanguineous, exudative fluid was obtained. Serum and pleural fluid levels of glucose, total protein, albumin, and lactose dehydrogenase are depicted in Table 1. Pleural fluid (PF) pH was not obtained. The cell count demonstrated 95% lymphocytes, 5% polymorphonuclear leucocytes, and no eosinophils. No microorganisms were seen on Gram’s stain and the cultures remained negative for microorganisms. The adenosine deaminase level in the PF was normal. *Mycobacterium tuberculosis* complex in BACTEC of PF was negative. *In vitro* lymphocyte transformation test (LTT) for cilazapril was not performed. Closed pleural biopsy (CPBx) revealed fibrous tissue with areas of calcification and occasional lymphocytes. Fifteen days later a repeat CXR revealed no improvement. Repeat CPBx remained nondiagnostic.

**Figure 2:**
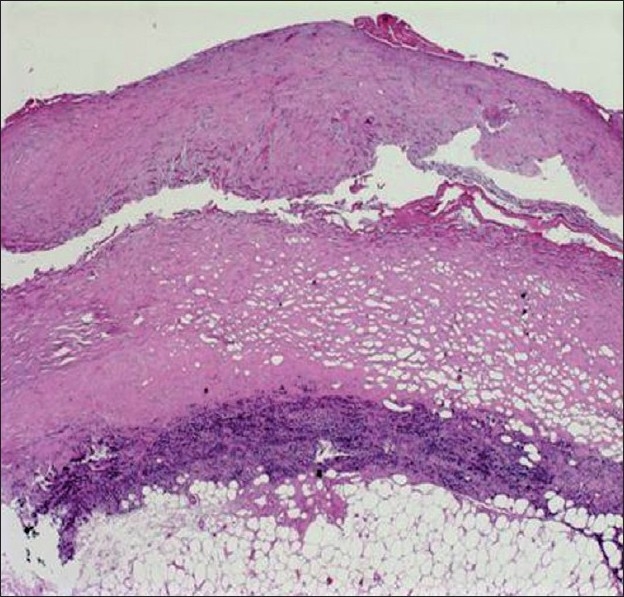
Pleural biopsy (from VATS) demonstrates thick hyalinized fibrinous tissue near the striated muscle and adipose tissue, with the infiltration of mononuclear inflammatory cells.

In view of his age, smoking history, and history of asbestos exposure, we wished to rule out an occult malignancy; we therefore performed a video-assisted thoroscopic surgery and multiple biopsies were obtained, which demonstrated thick hyalinized fibrinous tissue with mononuclear inflammatory cell infiltration [Figure 2]. No viral inclusion bodies were seen. BACTEC studies on pleural tissue remained negative.

**Figure 3:**
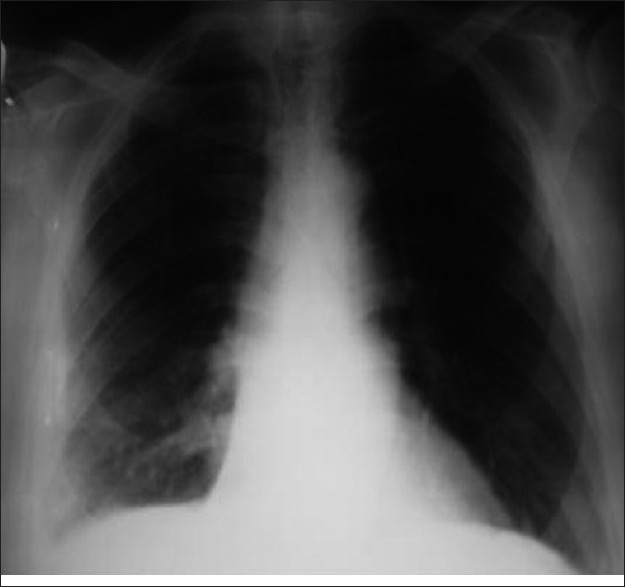
Posteroanterior chest X-ray after the cessation of cilazapril. The effusion has disappeared after the cessation of cilazapril

A few days after the surgery, the fluid reappeared. Cilazapril was discontinued and the PF disappeared dramatically [Figure 3]. Based on the collective findings, the diagnosis of ‘drug- induced pleural effusion due to ACEI’ was established. At present, the patient remains asymptomatic, without recurrence of PE.

**Table 1:** Serum and pleural biochemistry findings and their ratios according to Light’s criteria

	Pleura	Serum	Pleura/serum ratio
Glucose (mg/dl)	95	92	-
Total protein (g/dl)	4.52	8.7	0.5
LDH (U/l)	216	150	1.44

## Discussion

Adverse reactions to drugs produce only a small percentage of all PEs. In most cases the effusion rapidly resolves when the drug is discontinued. Hence, it is important to consider this possibility in all patients with PE before embarking on an extensive workup.

### Cilazapril-induced pleural effusion

There are a few drugs associated with eosinophilic PE; these are nitrofurantoin, dantrolene, bromocriptin, valproic acid, isotretionin, propylthiouracil, and ACEIs.[4] Hydralazine, penicillamine, procainamide, isoniazid, phenytoin, and chlorpromazine have been shown to produce a lupus-like syndrome, leading to PE.

Our search of the literature using the key words ‘pleural effusion,’ and ‘ACEI’ revealed only two articles.[2,4] Yoshida has reported a case of imidapril-induced eosinophilic PE with eosinophilia.[4] The diagnosis was established on clinical and laboratory grounds as well as by the response to withdrawal of the drug. Brunkhorst reported ramipril-induced polyserositis involving the pleura and the pericardium.[2] *In vitro* LTT results correlated with the clinical course and supported the theory of immune modulation. As far as we know, our case is only the third case report of ACEI-induced PE, and the first with cilazapril. The distinctive characteristic of our case is that the effusion was lymphocytic in nature, with no eosinophilia detected in the pleural fluid, blood, or lung tissue.

Bradykinin is one of the proinflammatory peptides that causes vasodilatation and increases vascular permeability and thus results in extravasation of protein and fluid.[5,6] In the carrageenan-induced rat model, ACE inhibition resulted in enhanced early production of endogenous bradykinin and edema formation.[7] Findings in experimental rat models[7,8] suggest that, in addition to hypersensitivity, bradykinin may play a role in the development of the pleurisy induced by ACEI. In the present case, there was no peripheral or PF eosinophilia to suggest that the hypersensitivity played a role in the formation of the fluid. However, we belive that, immune modulation might have been the causative factor. Our patient had used the ACEI for a short duration when his hypertension had first been detected. The pleural effusion appeared when he was prescribed the same medication again. This raises the possibility that the patient’s lymphocytes might have been sensitized by the drug during the initial course of the drug and produced the effusion on re-exposure. Apart from the fact that pleural fluid lymphocytosis was present and eosinophilia was absent, our hypothesis is also supported by Brunkhorst’s observation.[2]

Angioedema related to ACEI may lead to pleural involvement. Its pathophysiology involves immune modulation, elevated bradykinin levels, or complement-1-esterase deficiency;[2,9,10] however, that does not seem to be the scenario in our case.

Other causes that may result in lymphomonocytic pleural effusions are tuberculosis, cancer, post coronary arterial bypass grafting (CABG), viral pleurisy, lymphoma, sarcoidosis, rheumatoid disease, chylothorax, and asbestos exposure.[1] In our patient, these possibilities were well ruled out by the history, pleural biopsy, BACTEC studies, and PET scanning. He did not present with a ‘viral syndrome’ and had no symptoms for 6 weeks. Besides, the thickened pleura that was seen on the biopsy was in favor of a chronic process rather than an acute viral one.

The importance of this case report is that, it is the first case to be reported of a lymphocytic pleural effusion due to cilazapril (an ACEI). We arrived at the diagnosis by excluding the other possibilities. Even though a rechallenge test was not performed due to the ethical reasons, withdrawal of the drug led to rapid recovery, which is in favor of our diagnosis.

In summary, the possibility that cilazapril (an ACEI) may be responsible and should be considered in any patient with pleural effusion. The presence of lymphocytosis does not rule out drug-induced pleural effusion.
